# Structure–activity relationships of the N-terminus of calcitonin gene-related peptide: key roles of alanine-5 and threonine-6 in receptor activation

**DOI:** 10.1111/bph.12464

**Published:** 2013-12-23

**Authors:** Debbie L Hay, Paul WR Harris, Renata Kowalczyk, Margaret A Brimble, Dan L Rathbone, James Barwell, Alex C Conner, David R Poyner

**Affiliations:** 1 School of Biological Sciences and Maurice Wilkins Centre for Molecular Biodiscovery, The University of AucklandAuckland, New Zealand; 2 School of Chemical Sciences and Maurice Wilkins Centre for Molecular Biodiscovery, The University of AucklandAuckland, New Zealand; 3 School of Life and Health Sciences, Aston UniversityBirmingham, UK; 4 Medical School, University of BirminghamBirmingham, UK

**Keywords:** calcitonin gene-related peptide, efficacy, ligand binding, β-arrestin, cAMP, structure–activity relationship, GPCR, amylin

## Abstract

**Background and Purpose:** The N-terminus of calcitonin gene-related peptide (CGRP) is important for receptor activation, especially the disulphide-bonded ring (residues 1–7). However, the roles of individual amino acids within this region have not been examined and so the molecular determinants of agonism are unknown. This study has examined the role of residues 1, 3–6 and 8–9, excluding Cys-2 and Cys-7.

**Experimental Approach:** CGRP derivatives were substituted with either cysteine or alanine; further residues were introduced at position 6. Their affinity was measured by radioligand binding and their efficacy by measuring cAMP production in SK-N-MC cells and β-arrestin 2 translocation in CHO-K1 cells at the CGRP receptor.

**Key Results:** Substitution of Ala-5 by cysteine reduced affinity 270-fold and reduced efficacy for production of cAMP in SK-N-MCs. Potency at β-arrestin translocation was reduced by ninefold. Substitution of Thr-6 by cysteine destroyed all measurable efficacy of both cAMP and β-arrestin responses; substitution with either alanine or serine impaired potency. Substitutions at positions 1, 4, 8 and 9 resulted in approximately 10-fold reductions in potency at both responses. Similar observations were made at a second CGRP-activated receptor, the AMY_1(a)_ receptor.

**Conclusions and Implications:** Ala-5 and Thr-6 are key determinants of agonist activity for CGRP. Ala-5 is also very important for receptor binding. Residues outside of the 1–7 ring also contribute to agonist activity.

## Introduction

Calcitonin gene-related peptide (CGRP) is a 37 amino acid peptide, which in humans and rodents is found in two forms: α and β. It is an abundant neuropeptide that is widely distributed throughout the sensory nervous system. It is an extremely potent vasodilator, which is involved in neurogenic inflammation. CGRP receptor antagonists reduce migraine pain in clinical trials (Recober and Russo, 2009). CGRP forms a family with calcitonin (CT), adrenomedullin (AM), AM 2/intermedin, amylin and calcitonin receptor-stimulating peptide (CRSP) (Takei *et al*., 2004; Katafuchi *et al*., 2009).

These peptides act on the CT or CT receptor-like receptors (CLRs), which are family B GPCRs (receptor nomenclature follows Alexander *et al*., 2013). CLR in complex with the accessory protein receptor activity-modifying protein 1 (RAMP1) gives the CGRP receptor, whereas CLR with RAMP2 or 3 gives AM_1_ and AM_2_ receptors (receptor nomenclature follows Alexander *et al*., 2013; Supporting Information Table S1). The CT receptor complexes with RAMPs to give the AMY_1_, AMY_2_ and AMY_3_ receptors for amylin (McLatchie *et al*., 1998; Christopoulos *et al*., 1999; Muff *et al*., 1999); these exist as either (a) or (b) forms depending on the splice variant of CT receptor that is involved (Poyner *et al*., 2002). CGRP is a potent agonist at the AMY_1(a)_ receptor (Hay *et al*., 2005; Bailey *et al*., 2012).

The binding of CGRP to the CGRP receptor follows the typical pattern established for peptide ligands to family B GPCRs in that the C-terminus of the peptide binds to the extracellular domain of CLR (in association with the extracellular domain of RAMP1), whereas the N-terminus interacts with the transmembrane (TM) domain of CLR and its associated extracellular loops. It has been suggested that all the peptide ligands for family B GPCRs share a common motif at their N-termini: a helix cap or an activation loop (Neumann *et al*., 2008; Watkins *et al*., 2012). In the case of the CT/CGRP family, the motif is formed by a disulphide-bonded ring; in CGRP this involves cysteines at positions 2 and 7. Truncation of the disulphide-bonded loop gives the antagonist, CGRP_8–37_, showing that residues 1–7 are required for receptor activation and N-terminal fragments of CGRP are reported to have agonist activity (Chiba *et al*., 1989; Maggi *et al*., 1990).

There has been some work on the structure–activity relationship for the C-terminus of CGRP acting on the CGRP receptor (Conner *et al*., 2002; Lang *et al*., 2006), but much less is known about the N-terminus of the peptide when it activates this receptor or the AMY_1_ receptor (Watkins *et al*., 2013). The importance of the disulphide bond has been established (Dennis *et al*., 1989; Saha *et al*., 1998), and there are a few studies based mainly on deletions (Thiebaud *et al*., 1991; Hakala *et al*., 1994; Heino *et al*., 1998). It is possible to extend the N-terminus of CGRP, as with [Tyr^0^]-CGRP, while retaining high affinity (Dennis *et al*., 1989) although efficacy may be compromised (Poyner *et al*., 1992). The details of how CGRP interacts with the TM and extracellular loop regions of its receptor(s) remain obscure (Barwell *et al*., 2011b).

Although there is little experimental evidence for the role of individual amino acids at the N-terminus of CGRP, comparison of naturally occurring peptide sequences is informative. Position 1 of CGRP is not fully conserved and can either be serine or alanine (Supporting Information Fig. S1). Position 3 is usually asparagine, although in human α and marmoset CGRP, it is aspartate. Thereafter, there is a very high degree of sequence identity up to position 14 in species from mammals to bony fish, emphasizing the conservation at the N-terminus of CGRP. Comparing CGRP with other members of the CT family of peptides including examples of the primitive cartilaginous and jawless fish (Supporting Information Fig. S2; see also Wong and Takei, 2009; Takei *et al*., 2010), the most striking feature is the conservation of position 6 in peptides apart from equine CGRP-1 [actually the equine equivalent of CRSP (Ogoshi *et al*., 2006)], CRSP 2 and 3 and AM4 in *Takifugu rubripes* and *Tetraodon nigroviridis*. AM4 has only been described in bony fish and in other species the threonine is retained. The equivalent of positions 1 and 5 is group conserved as small hydrophobic amino acids (glycine, alanine and serine). There is extensive conservation of residues 1–9 between CGRP and amylin from all species.

In this study, we investigated the structure–activity relationship of the N-terminus of CGRP, using amino acid substitutions of residues 1, 3–6 and 8–9, on CGRP receptor binding, production of cAMP and β-arrestin 2 translocation. These positions were substituted with either alanine or cysteine. Cysteine and alanine are small hydrophobic amino acids that are often group conserved along with serine in proteins. The results indicate that Ala-5 and Thr-6 are important determinants of CGRP activity at the CGRP receptor.

## Methods

### Peptides

All peptides are derivatives of human α CGRP, containing a Cys-2-Cys-7 disulphide bond and C-terminal amide. CGRP was purchased from American Peptide (Sunnyvale, CA, USA) or Bachem (St Helens, Merseyside, UK). [Cys^1^]-, [Cys^6^]-, [Cys^8^]- and [Cys^9^]-CGRP were synthesized by Alta Biosciences, Birmingham, UK. [Cys^3^]-, [Ala^4^]-, [Cys^5^]-, [Ala^6^]-, [Ser^6^]-, [Asp^6^]-, [Lys^6^]-, [Ala^8^]- and [Ala^9^]-CGRP peptides were synthesized by solid phase peptide synthesis at the University of Auckland using the Fmoc/^t^Bu method on a 0.1 mmol scale. Briefly, Rink amide aminomethyl resin was prepared as described (Harris *et al*., 2011), and the peptide elongated using a CEM microwave peptide synthesizer (CEM Corporation, Matthews, NC, USA) as previously described (Harris *et al*., 2008). For [Cys^3^]- and [Cys^5^]-CGRP peptides, the non-disulphide cysteines were protected as the trifluoroacetic acid-stable *tert*-butyl ethers. For the other cysteine-substituted peptides, the non-disulphide cysteines were protected as acetamidomethyl derivatives. The peptides were cleaved from the resin with concomitant removal of side chain protecting groups with 94.0% trifluoroacetic acid, 1.0% triisopropylsilane, 2.5% water and 2.5% ethanedithiol (v/v/v/v) for 2–3 h, precipitated with cold diethyl ether, isolated by centrifugation, dissolved in 50% aqueous acetonitrile containing 0.1% trifluoroacetic acid and lyophilized. The crude peptides were dissolved in 0.1 M Tris (pH = 8.3) at a concentration of 1 mg·mL^−1^ and the oxidation (disulphide formation) allowed to proceed at room temperature open to air. Monitoring by reverse phase HPLC indicated that the reaction was typically complete after 12 h. The crude product was lyophilized, redissolved in 50.0% aqueous acetonitrile containing 0.1% trifluoroacetic acid and purified by semi-preparative reverse phase HPLC using a C18 Gemini (Phenomenex, Torrance, CA, USA) column (10 × 250 mm) at a flow rate of 5 mL·min^−1^ and eluted using an appropriate gradient based on the analytical HPLC profile. Fractions containing the pure peptide were identified by electrospray mass spectrometry and/or HPLC, pooled and lyophilized. All peptides were >95% purity as judged by integration of the HPLC chromatogram at 210 nm, and peptide masses were confirmed by electrospray mass spectrometry. For [Cys^3^]- and [Cys^5^]-CGRP peptides, following the oxidation, the crude peptide was recovered by solid phase extraction and lyophilized. To remove the *tert*-butyl on the cysteine at [Cys^3^] or [Cys^5^], the peptide was then dissolved in trifluoroacetic acid : anisole (9:1, v/v) at a concentration of 15 mg·mL^−1^, cooled to 0°C and trifluoroacetic acid : trifluoromethanesulphonic acid (4:1, v/v) (0.4 mL) was added. The solution was stood at 0°C for 3 min, poured into cold ether and recovered by centrifugation. Purification as described earlier afforded the pure peptides in >95% purity.

### Cell culture and transfection

SK-N-MC cells were grown in DMEM/F12 medium supplemented with 10% fetal calf serum as described previously (Poyner *et al*., 1998). Cos 7 cells were grown in DMEM/10% FBS and were transfected with human HA-tagged CT (a) receptors and human myc-tagged RAMP1 using polyethylenimine and identical methods to those described previously for the CGRP receptor (Bailey and Hay, 2006). In this case, the transfection gives an AMY_1(a)_ receptor. This protocol has been successfully used to express these receptors (Qi *et al*., 2008). This protocol was also used to express the CGRP receptor in Cos 7 where appropriate.

### Measurement of cAMP

Production of cAMP in SK-N-MC cells for all analogues except [Cys^5^]-hαCGRP was by a radioreceptor assay, as described previously (Poyner *et al*., 1998). Cells were treated with increasing concentrations of the CGRP analogues for approximately 10 min. Where antagonist activity was being assessed, the cells were pretreated with peptides for 10 min prior to the addition of the CGRP derivative (Poyner *et al*., 1998). For [Cys^5^]-hαCGRP, a different clone of SK-N-MC cells was used and cAMP production was measured via AlphaScreen (Perkin Elmer, Wellesley, MA, USA), as described previously (Gingell *et al*., 2010). This assay was also used to investigate the action of all derivatives at the AMY_1(a)_ receptor and other experiments using Cos 7 cells. In control experiments to confirm cross-compatibility of the two SK-N-MC cell clones using AlphaScreen, [Ala^4^]-hαCGRP and [Ala^6^]-hαCGRP gave similar reductions in pEC_50_ values as seen when this parameter was measured by a radioreceptor assay with the other clone of cells (values of 7.58 ± 0.43 and 7.39 ± 0.16 vs. 9.00 ± 0.33 for CGRP, *n* = 3–4; compare with Table 1).

**Table 1:** pK_i_, pEC_50_ and *E*_max_ values for CGRP analogues on SK-N-MC and CHO-K1 cells

	Binding (SK-N-MC)			cAMP (SK-N-MC)			β-Arrestin (CHO-K1)		
	pK_i_		pEC_50_		*E*_max_ (% CGRP)	pEC_50_		*E*_max_ (% CGRP)
	Control	CGRP analogue	Control	CGRP analogue	CGRP analogue	Control	CGRP analogue	CGRP analogue
[Cys^1^]	10.29 ± 0.05	9.55 ± 0.20**	8.80 ± 0.09	7.70 ± 0.28*	71 ± 8*	9.20 ± 0.16	8.58 ± 0.05*	60 ± 1**
[Cys^3^]	10.29 ± 0.05	9.54 ± 0.07**	8.80 ± 0.09	8.27 ± 0.11	116 ± 4	9.20 ± 0.16	8.43 ± 0.05**	95 ± 3
[Ala^4^]	10.29 ± 0.05	9.40 ± 0.42	8.80 ± 0.09	7.43 ± 0.26**	100 ± 11	9.20 ± 0.16	8.42 ± 0.04**	73 ± 3*
[Cys^5^]	10.43 ± 0.20	8.00 ± 0.26***	9.43 ± 0.07	7.18 ± 0.18†	23 ± 4†	9.20 ± 0.16	8.25 ± 0.04***	55 ± 2**
[Ala^6^]	9.99 ± 0.17	8.57 ± 0.49*	8.48 ± 0.10	6.59 ± 0.20**	72 ± 12	9.20 ± 0.16	7.74 ± 0.28*	43 ± 2.0***
[Cys^6^]	10.29 ± 0.05	9.33 ± 0.13**	–	Undetectable	Undetectable	–	Undetectable	Undetectable
[Ser^6^]	9.99 ± 0.17	9.12 ± 0.54*	8.48 ± 0.10	7.27 ± 0.11*	100 ± 10	9.20 ± 0.16	8.07 ± 0.23*	57.3 ± 4.8**
[Asp^6^]	9.99 ± 0.17	7.50 ± 0.22**	–	Undetectable	Undetectable	–	Undetectable	Undetectable
[Lys^6^]	9.99 ± 0.17	7.76 ± 0.64**	–	Undetectable	Undetectable	–	Undetectable	Undetectable
[Ala^8^]	9.99 ± 0.17	9.42 ± 0.42	8.48 ± 0.10	7.73 ± 0.25**	104 ± 8	9.20 ± 0.16	8.81 ± 0.04	81 ± 3*
[Ala^9^]	9.99 ± 0.17	9.28 ± 0.20	8.48 ± 0.10	7.30 ± 0.08**	103 ± 13	9.20 ± 0.16	8.23 ± 0.08**	64 ± 3**

Values are means ± SEM, *n* = 3–6.

*,**,*** *P* < 0.05, 0.01, 0.001 compared with CGRP, Dunnett's test following one-way anova.

† No response seen in five experiments.

### β-Arrestin translocation

Translocation of β-arrestin after a 2 h stimulation with peptides was measured using a DiscoveRx ‘PathHunter eXpress β-arrestin’ kit (93-0446E; Birmingham, West Midlands, UK), with CHO-K1 cells expressing the human CGRP receptor and β-arrestin 2. The role of β-arrestin 2 in CGRP receptor desensitization has been previously demonstrated (Hilairet *et al*., 2001; Padilla *et al*., 2007).

### Radioligand binding

The ability of peptides to displace ^125^I-CGRP binding to SK-N-MC cell membranes was measured in a microcentrifugation-based binding assay, as described previously (Poyner *et al*., 1998).

### Data analysis

GraphPad Prism 5.00 or 6.00 (GraphPad Software Inc., San Diego, CA, USA) was used for data fitting. Concentration–response curves were fitted to a sigmoidal function with Hill slope constrained to unity to obtain pEC_50_ and maximum responses (*E*_max_) values. Displacement curves were analysed to obtain pIC_50_ values. Schild plots were fitted by linear regression to obtain slope and intercepts. pEC_50_ and pIC_50_ values were compared with that of human αCGRP using Dunnett's test with either a normal or repeated measures one-way anova, as appropriate. To assess if *E*_max_ values differed from wild type, 95% confidence limits were calculated using Student's *t*-statistic.

### Materials

[^125^I]-iodohistidyl^8^ human α CGRP (^125^I-CGRP) and [^3^H]-cAMP were purchased from Perkin Elmer (Wellesley, MA, USA). Other reagents were as described previously (Barwell *et al*., 2011a). The SK-N-MC cells were from the European Collection of Animal Cell Cultures (Porton Down, UK), except those used for examining [Cys^5^]-CGRP, which were a gift from Dr Fiona Marshall (Heptares, Stevenage, UK) (used for radioligand binding) or from American Type Culture Collection (used for cAMP measurements). Cells were changed due to problems with the old batch losing expression of CGRP receptors over passages, as has been documented previously (Choksi *et al*., 2002).

## Results

### The role of the side chains of positions 1, 3, 4, 8 and 9 at CGRP receptors

**Figure 1:**
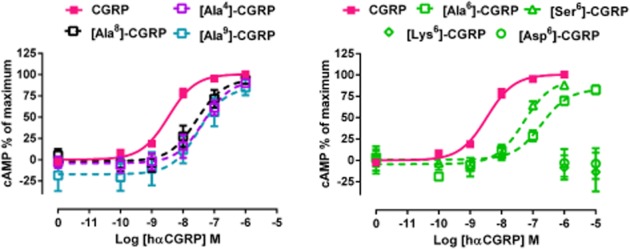
Effects of CGRP alanine and Thr-6 analogues on cAMP production in SK-N-MC cells. Values are means ± SEM, *n* = 3–6 and are normalized to the response seen with CGRP.

As expected, CGRP was a potent agonist at stimulating cAMP production in SK-N-MC cells (Figure 1; Table 2), with a pEC_50_ within the considerable range reported in the literature (Poyner *et al*., 1998; Howitt *et al*., 2003); it also displaced ^125^I-CGRP with a Ki that was 30-fold less than its pEC_50_ (Figure 3; Table 2). It was a very potent agonist at stimulating β-arrestin 2 translocation in CHO-K1 cells (Figure 4; Table 2006).

**Figure 2:**
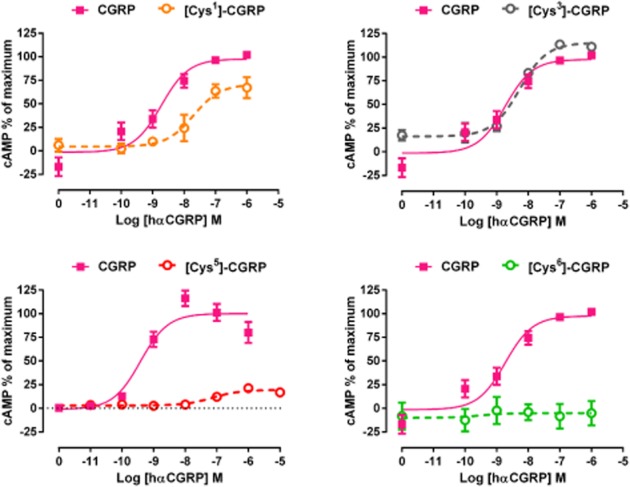
Effects of CGRP cysteine analogues on cAMP production in SK-N-MC cells. Values are means ± SEM, *n* = 3–6 and are normalized to the response seen with CGRP.

**Figure 3:**
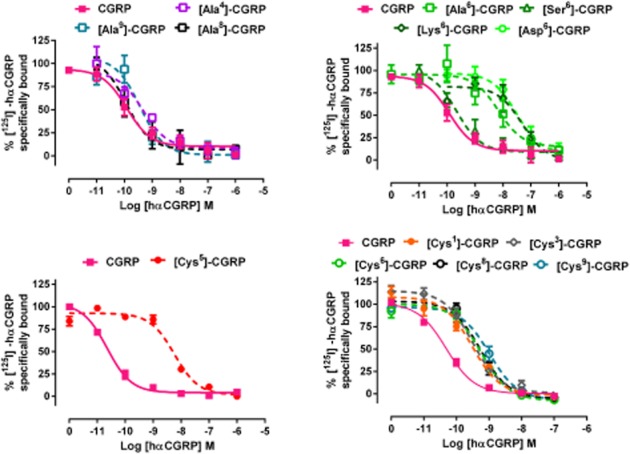
Inhibition of [^125^I]-CGRP binding by CGRP analogues in SK-N-MC cell membranes. Values are means ± SEM, *n* = 3–6.

As can be seen in Table 2006 and Figures 2 and 3, the introduction of a cysteine to replace aspartate in native CGRP at position 3 made little difference to potency on cAMP although there was a small (approximately sixfold) decrease in affinity. Substitution of alanine by cysteine at position 1 caused around a 10-fold decrease in potency on cAMP and modest reduction in *E*_max_; these changes were accompanied by a sixfold decrease in affinity. Substitution of Thr-4, Val-8 and Thr-9 in native CGRP by alanine caused 6- to 10-fold decreases in potency on cAMP; there were no significant changes in affinity.

**Figure 4:**
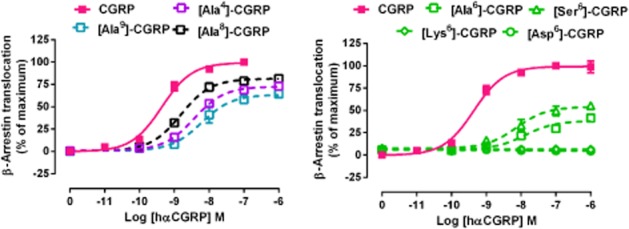
Stimulation of β-arrestin 2 translocation by CGRP alanine and Thr-6 analogues in CHO-K1 cells. Values are means ± SEM, *n* = 3 and are normalized to the response seen with CGRP.

**Figure 5:**
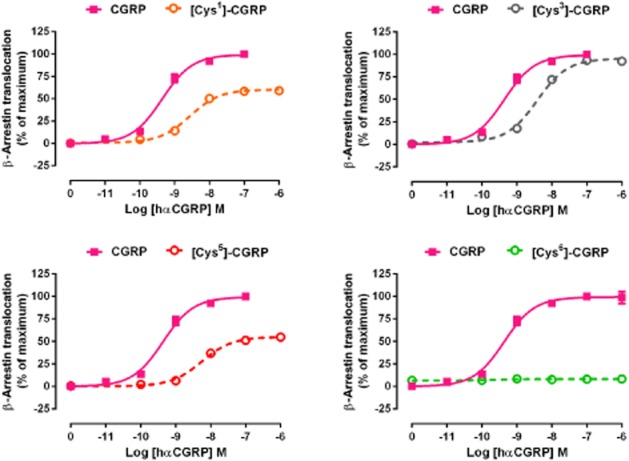
Stimulation of β-arrestin 2 translocation by CGRP cysteine analogues in CHO-K1 cells. Values are means ± SEM, *n* = 3 and are normalized to the response seen with CGRP.

The analogues were also examined for their activity at stimulating β-arrestin 2 translocation in CHO-K1 cells (Figures 4 and 5; Table 1). They all showed a reduced potency of 3- to 10-fold, which was significant for every analogue except [Ala^8^]-CGRP. Apart from [Cys^3^], they also all showed decreased *E*_max_; for [Ala^8^]-CGRP the reduction in maximum was 20%, but for the other analogues it was approximately 40%.

### The role of the side chains of positions 5 and 6

**Figure 6:**
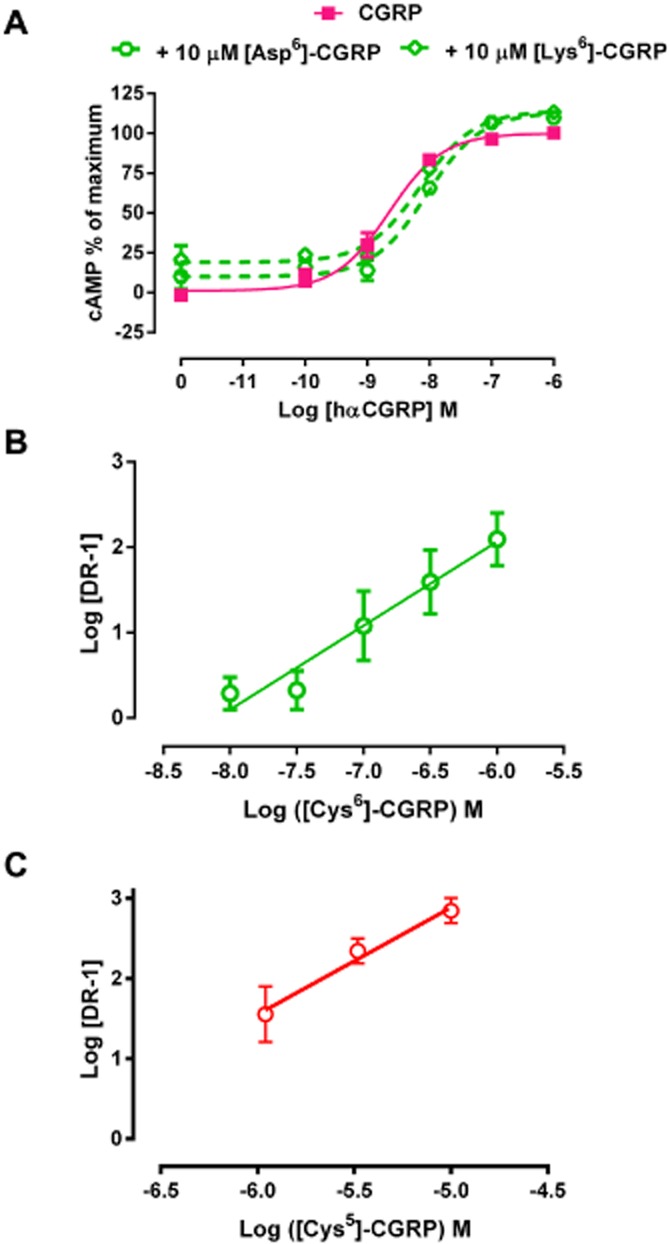
Antagonism of cAMP production by CGRP with (A) [Asp^6^] and [Lys^6^]- and (B) [Cys^6^]-CGRP, (C) [Cys^5^]-CGRP in SK-N-MC cells.

Substitution of Ala-5 in native CGRP by cysteine caused over a 270-fold decrease in affinity. There was a ninefold decrease in potency for stimulation of β-arrestin 2 translocation, and the *E*_max_ was reduced by 45% compared with CGRP. The analogue was a partial agonist at stimulating cAMP production in SK-N-MC cells. A measureable response was only found in cells where the pEC_50_ for CGRP was over 9, indicating tight coupling to G_s_ activation. In these cells, the *E*_max_ was reduced by almost 70%, and the potency was decreased over 100-fold (Table 2006; Figures 2, 3 and 5). Consistent with its low efficacy, it could antagonize the action of CGRP. The slope of the resulting Schild plot was not significantly different from unity (1.33 ± 0.30), resulting in a pK_b_ estimate for [Cys^5^]-CGRP of 7.74 ± 0.12 (Figure 6C). This is in good agreement with the pK_i_ of 8.00 ± 0.26 (Table 2006). By comparison, in the same series of experiments, CGRP_8–37_, the best characterized CGRP antagonist, had a pK_b_ of 9.42 ± 0.12 (*n* = 4, data not shown). Although this is rather high, considerable variations in affinity for this antagonist are sometimes seen (Hay *et al*., 2008).

Substitution of Thr-6 in native CGRP by either alanine or serine caused substantial decreases in potency in cAMP production; the potency for [Ser^6^]-CGRP was reduced over 10-fold and for [Ala^6^]-CGRP there was almost a 100-fold reduction in potency with a trend towards a reduced *E*_max_. There were reductions in affinity that was the largest for [Ala^6^]-CGRP (30-fold). There were also decreases in potency when β-arrestin 2 translocation was examined and both the analogues were partial agonists, with the *E*_max_ to [Ser^6^]-CGRP reduced by 40% and that to [Ala^6^]-CGRP by almost 60% (Figure 4; Table 2006).

Substitution of Thr-6 by cysteine, aspartate and lysine had more profound effects. [Asp^6^]- and [Lys^6^]-CGRP both had over 100-fold decreases in affinity. They also failed to stimulate cAMP production or β-arrestin 2 translocation at concentrations of up to either 1 μM (β-arrestin 2) or 10 μM (cAMP). The loss of efficacy was confirmed by examining the cAMP response to CGRP in the presence of 10 μM of either of these agents. Both produced small but significant rightward shifts in the concentration–response curves to CGRP with no suppression of the *E*_max_ (Figure 6A): pEC_50_ for CGRP, 8.66 ± 0.19; with 10 μM [Asp^6^]-CGRP, 8.04 ± 0.04 (*P* < 0.05) and with 10 μM [Lys^6^]-CGRP, 8.11 ± 0.09 (*P* < 0.01), indicating that they were acting as competitive antagonists. For [Cys^6^]-CGRP, the loss in affinity was only fivefold (Figure 3; Table 2006), but as with the aspartate and lysine derivatives, it was also unable to stimulate either production of cAMP or translocation of β-arrestin 2. To confirm the loss of efficacy at cAMP production, the analogue was used as an antagonist to shift the concentration–response curve to CGRP (Figure 6B); the Schild plot had a straight line with a slope not significantly different from unity (0.98 ± 0.20) and a pA_2_ of 8.08 ± 0.13 (*n* = 3). This indicates that it behaves as a competitive antagonist.

### Actions of analogues at stimulating cAMP production at the AMY_1(a)_ receptor

**Figure 7:**
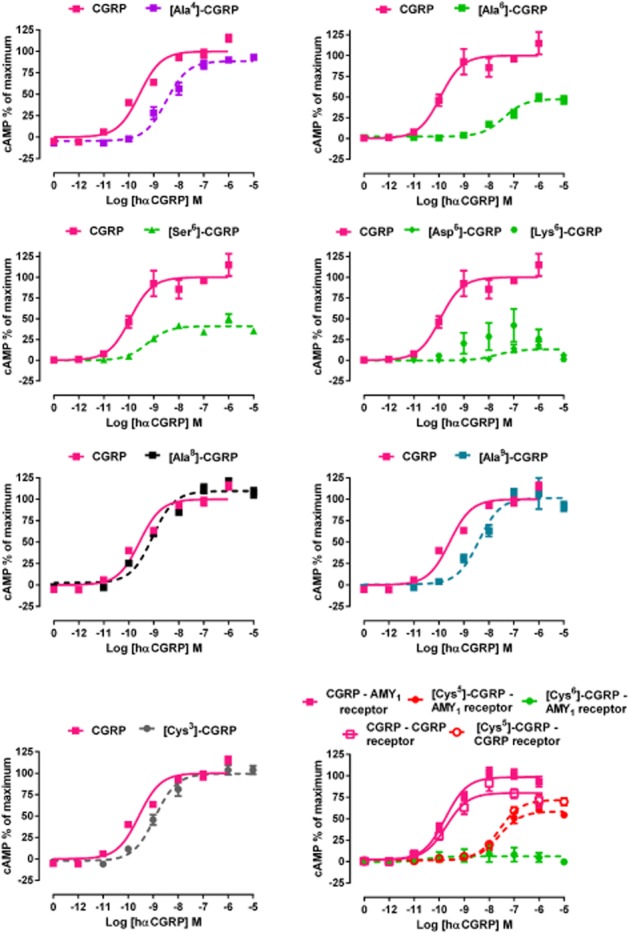
Effects of CGRP analogues on cAMP production in AMY_1(a)_ receptors in Cos 7 cells. A single experiment representative of 3–6 is shown. Each point is the mean ± SEM of three or four determinations. Responses are normalized to the response seen with CGRP. For the final part of this figure, the response of CGRP and [Cys^5^]-CGRP on the CGRP receptor transfected into these cells is shown alongside the AMY_1(a)_ receptor data.

**Figure 8:**
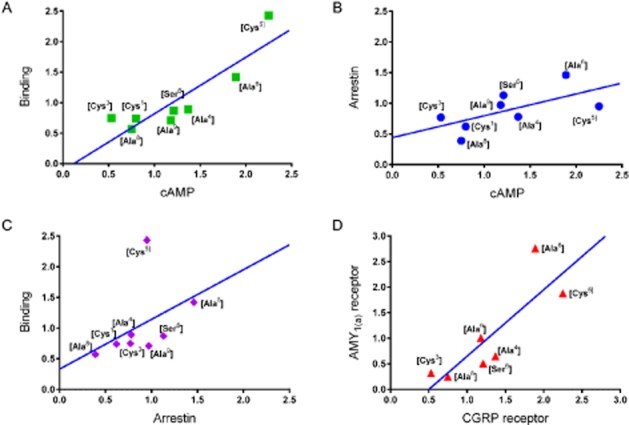
Correlations between effects of CGRP analogues. (A) Binding v cAMP production, CGRP receptor; (B) beta-arrestin translocation v cAMP production, CGRP receptor; (C) binding v beta-arrestin translocation, CGRP receptor; (D) cAMP production, AMY1(a) receptor v CGRP receptor. Values are the Δlog pEC_50_, or Δlog pK_i_, where the value for the analogue was subtracted from that of its paired control. Thus, 1 represents a 10-fold reduction, 2 represents a 100-fold reduction and 3 for 1000-fold. Where activity could not be detected no value is included.

The structure–activity relationship with the analogues at the CGRP receptor was also broadly apparent when the derivatives were used to stimulate cAMP production in Cos 7 cells via the AMY_1(a)_ receptor ( Table 2012; Figures 7 and 8), which uses the CT receptor rather than CLR. [Cys^5^]-CGRP was 75-fold less potent than CGRP and was a partial agonist. In Cos 7 cells transfected with CLR and RAMP1 (Figure 7), [Cys^5^]-CGRP appeared as a full agonist (*E*_max_ 90 ± 8% of that of CGRP) albeit much less potent than CGRP itself (pEC_50_ for CGRP, 9.66 ± 0.22; pEC_50_ for [Cys^5^]-CGRP, 7.90 ± 0.20). [Ala^9^]-hαCGRP was 10-fold less potent than CGRP. [Ala^6^]-CGRP was over 700-fold less potent than CGRP and it was also a partial agonist. There were no consistent stimulations of cAMP production seen with [Cys^6^]-, [Asp^6^]- or [Lys^6^]-CGRP. The potency of [Ser^6^]-CGRP was not significantly different from that of CGRP, and although there was a trend for a reduced *E*_max_, this did not reach significance and [Ala^8^]-CGRP also behaved like CGRP.

**Table 2:** Actions of CGRP analogues on AMY_1(a)_ receptor-stimulated cAMP production in Cos 7 cells

	pEC_50_		*E*_max_ (% CGRP)
	Control	CGRP analogue	CGRP analogue
[Cys^3^]	9.22 ± 0.13	8.90 ± 0.03	108 ± 4
[Ala^4^]	9.22 ± 0.13	8.57 ± 0.22*	161 ± 45
[Cys^5^]	9.66 ± 0.15	7.78 ± 0.12***	50 ± 4***
[Ala^6^]	9.76 ± 0.15	7.00 ± 0.44**	42 ± 11**
[Cys^6^]	–	Undetectable			
[Ser^6^]	9.76 ± 0.15	9.25 ± 0.03	76 ± 19
[Asp^6^]	–	Undetectable			
[Lys^6^]	–	Undetectable			
[Ala^8^]	9.22 ± 0.13	8.97 ± 0.07	143 ± 11
[Ala^9^]	9.22 ± 0.13	8.21 ± 0.32**	89 ± 9

Values are means ± SEM, *n* = 3–6.

*,**,*** , *P* < 0.05, 0.01, 0.001 compared with CGRP, Dunnett's test followed by one-way anova or Student's *t*-test, as appropriate.

## Discussion

This paper assesses the contribution of the first nine residues of CGRP, excluding the cysteines at positions 2 and 7 to receptor binding and activation. The results highlight the contribution of residues in the middle and C-terminal portions of this part of CGRP, especially Ala-5 and Thr-6.

Substitution of the alanine at position 5 by cysteine causes large decreases in affinity and efficacy at cAMP production in SK-N-MC cells (Figure 8). The affinity loss is large, especially considering that there is only around a 10-fold loss typically found for deletion of the first seven amino acids to give CGRP_8–37_ (Chiba *et al*., 1989; Watkins *et al*., 2013). It seems that the sulfhydryl group at position 5 results in the N-terminus adopting a conformation that impairs the binding of the rest of the CGRP molecule. In CHO-K1 cells, the reduction in efficacy seen with [Cys^5^]-CGRP at stimulating β-arrestin translocation is smaller than that seen on cAMP in SK-N-MC cells, although this may simply reflect the better receptor coupling seen in the former cells, as discussed below.

Substitution of Thr-6 by cysteine gave only a modest reduction in affinity, but all measurable efficacy was lost for coupling to both Gs and translocation of β-arrestin 2. Threonine is able to take part in both hydrogen bonds and hydrophobic interactions, and the structure–activity relationship revealed in this study suggests that both are likely to be important. The failure of serine to substitute for threonine at CGRP is striking as the position of the hydroxyl group in the two analogues is identical; they differ only in that threonine has an extra methyl group on the β-carbon. However, these data are consistent with the fact that serine is not found in position 6 in any known native member of the CT/CGRP family (Supporting Information Figs S1 and S2). It would appear likely that the threonine fits into a tightly constrained pocket, where both the methyl and the hydroxyl groups are important. The probability is that the hydroxyl group is involved in a hydrogen bond; the methyl group may pack against a hydrophobic group. The poor binding and lack of efficacy seen with both the lysine and aspartate derivatives may imply that the partner for threonine is uncharged; however, it is also possible that steric hindrance could explain the large effects with these derivatives.

Throughout the CT/CGRP family, positions 5 and 6 are highly conserved, suggesting that they may have similarly important roles across all members of the family. The equivalent of position 5 is group conserved as a small hydrophobic (or weakly hydrophilic) residue: alanine, serine or glycine, except for AM4 sequences where larger hydrophobic residues are present. The equivalent of position 6 is always a threonine except in AM4 and CRSP 2 and 3, and there is some doubt whether these three peptides are agonists at CLR-based receptors. Porcine CRSP 2 and 3 do not stimulate cAMP production at CLR or CT receptor expressed in Cos 7 cells, with or without RAMPs (Katafuchi *et al*., 2009), and dog CRSP 2 has no action on the CT receptor of LLC-PK cells (Ogoshi *et al*., 2006). There is no information on the biological activity of AM4, although as the mRNA is present in large amounts in the skin of *Takifugu*, it has been suggested that it may have an antimicrobial role (Ogoshi *et al*., 2003).

There is no previous work on position 5 of CGRP, but one study has looked at position 6. [Val^6^]-CGRP was inactive at stimulating cAMP production in porcine iris ciliary body (Heino *et al*., 1998), in agreement with the conclusions from this study that the residue cannot be substituted without loss of activity. Interestingly, an AM derivative, where replacement of the equivalent of Thr-6 (Thr-20) was reported to reduce potency on blood pressure (Kuwasako *et al*., 2011), is consistent with this being an important residue for all members of the CT/CGRP family. Similarly, substitution of Thr-6 in amylin by alanine resulted in reduced activity of the peptide (Roth *et al*., 2008).

Substitution of the remaining residues of CGRP had generally rather modest but significant effects on binding and potency at stimulating cAMP and β-arrestin 2 translocation. There was a good correlation between the potency of the analogues at stimulating cAMP and their binding affinities (*r*^2^ = 0.78), although the correlation between binding and β-arrestin translocation was much weaker (*r*^2^ = 0.40, Figure 8). Asp-3 was the least significant substitution with cysteine only causing a small reduction in affinity. A photoaffinity probe can be accommodated here with retention of high affinity binding (Stangl *et al*., 1993), consistent with the side chain of the residue sitting in an exposed area when CGRP is bound to its receptor, although it is highly conserved in CGRP as either aspartate or asparagine. Substitutions at all the remaining positions impaired the potency of the analogue at stimulating cAMP and efficacy at β-arrestin 2 translocation, indicating that their side chains have roles in receptor activation. There was little evidence for selective activation of either of the two pathways with any of these analogues (Figure 8), although as the responses were compared in different cell lines, quantitative comparison of efficacy measures is difficult.

As CGRP_8–37_ is an antagonist, it is surprising that substitutions at positions 8 and 9 apparently reduced efficacy. However, a photoaffinity probe attached to position 8 of salmon CT can label a residue at the top of the third extracellular loop (Dong *et al*., 2004). Potentially, residues in this part of the ligand could interact directly with the juxtamembrane part of the receptor and enhance the ability of the disulphide-bonded ring to cause receptor activation. Thr-9 may, alternatively, act indirectly by helping to stabilize a conformation of the N-terminus of CGRP that is favourable for binding by making a hydrogen bond to connect with an appropriate H-bond acceptor located between residues 1 and 7 on the disulphide-bonded ring. There is support for this from NMR evidence (Breeze *et al*., 1991) and molecular dynamics simulations (D. L. Rathbone and D. R. Poyner, unpublished) as well as the position of this residue within the N-cap proposed by Watkins *et al*. (2012). Whatever the mechanism is, it is clear that agonist activity is influenced by residues outside the disulphide-bonded loop. Interestingly, CGRP_1–7_ is reported to be an antagonist (Dennis *et al*., 1989) whereas CGRP_1–12_ can mimic the hypotensive effects of CGRP and so may be an agonist (Maggi *et al*., 1990); this raises the possibility that the 1–7 ring requires other residues such as Val-8 and Thr-9 in order to activate the receptor.

The CGRP analogues showed a similar pattern of activity for stimulation of cAMP production on the AMY_1(a)_ receptor as they did on the CGRP receptor (Figure 8D; *r*^2^ = 0.70); CGRP is a potent agonist at both receptors (Hay *et al*., 2005). In particular, Ala-5 and Thr-6 were both important for agonist action. Potency was also influenced by substitution of Thr-9; this is conserved between CGRP and amylin. Thus, the same general determinants of receptor activity are likely to operate at both receptors for coupling to Gs. A detailed comparison of the importance of individual residues at stimulating the AMY_1(a)_ and the CGRP receptor is complicated by the fact that the AMY_1(a)_ receptor was expressed in Cos 7 cells and the CGRP receptor was expressed in SK-N-MC cells. There seems to be better coupling of the CGRP receptor to Gs in the Cos 7 cells compared with the SK-N-MC cells as [Cys^5^]-CGRP was a full agonist in the former but not the latter. This could also relate to relative expression levels of the receptors (endogenous vs. overexpressed).

Although it is not possible on the basis of the data in this study to propose a model of how CGRP binds to its receptor, a number of general points can be made. The first and third extracellular loops of CLR play only relatively minor roles in binding CGRP (Barwell *et al*., 2011a); the second extracellular loop is much more significant (Barwell *et al*., 2012; Woolley *et al*., 2013). We have previously modelled CGRP bound to the TM domain of CLR, in close proximity to CLR (Woolley *et al*., 2013). This model is consistent with the data in the current study. The side chain of Ala-5 and the methyl group of Thr-6 are buried in the interface between TM helices 5 and 6; the hydroxyl group on Thr-6 could H-bond to a backbone carbonyl on the C-terminal portion of the second extracellular loop. Asp-3 is in an unhindered position, free from steric constraints. Ala-1 points between the top of the first extracellular loop and TM helix 1, allowing N-terminal extensions of the peptide. The residues that interact with CGRP in the CLR as part of the CGRP receptor are likely to be conserved in the CT receptor.

In conclusion, this paper provides new information on the structural requirements needed for agonist activity of CGRP. Alanine at position 5 and threonine at position 6 are particularly important for receptor activation. CGRP positions 1, 4, 8 and 9 also influence agonist activity. It would be interesting to evaluate analogues based on the pentapetide CGRP_5–9_, as this may contain the key components needed for receptor activation.

## Abbreviations

AM: adrenomedullin
CGRP: calcitonin gene-related peptide
CLR: calcitonin receptor-like receptor
CRSP: calcitonin receptor-stimulating peptide
CT: calcitonin
*E*_*max*_: maximum response
RAMP: receptor activity-modifying proteins

## Supporting Information

**Figure S1** Alignment of CGRP sequences. **Figure S2** N-terminal regions of CGRP/calcitonin family members, aligned over CGRP_1–20_. **Table S1** Pharmacology of selected CLR/RAMP and CTR/RAMP complexes.
