# Supplementary material for: Structure–activity relationships of the N-terminus of calcitonin gene-related peptide: key roles of alanine-5 and threonine-6 in receptor activation
Source: Br J Pharmacol. 2013 Dec 23;171(2):415–26. doi: 10.1111/bph.12464 (PMC3904261; doi:10.1111/bph.12464)
Supplement: Supplementary file 1 — Figure S1 Alignment of CGRP sequences. Figure S2 N-terminal regions of CGRP/calcitonin family members, aligned over CGRP1–20. Table S1 Pharmacology of selected CLR/RAMP and CTR/RAMP complexes. [file bph0171-0415-sd1.docx]

Supplementary Figure 1: Alignment of CGRP sequences.

ACDTATCVTHRLAGLLSRSGGVVKNNFVPTNVGSKAF Human alpha

ACNTATCVTHRLAGLLSRSGGMVKSNFVPTNVGSKAF Human beta

SCNTATCVTHRLAGLLSRSGGVVKDNFVPTNVGSEAF Rat alpha

SCNTATCVTHRLAGLLSRSGGVVKDNFVPTNVGSKAF Rat beta

ACDTATCVTHRLAGLLSRSGGMVKNNFVPTNVGSEAF Marmoset

SCNTATCVTHRLAGLLSRSGGVVKDNFVPTNVGSEAF Mouse

SCNTATCVTHRLAGLLSRSGGMVKSNFVPTDVGSEAF Pig

SCNTATCVTHRLAGLLSRSGGVVKNNFVPTNVGSEAF Dog

SCNTATCVTHRLAGLLSRSGGVVKSNFVPTNVGSEAF Bovine

SCNTATCVTHRLAGLLSRSGGVVKSNFVPTDVGSEAF Horse

GCNTATCVTHRLADFLSRSGGVAKSDFVPTNVGAKAF short-tailed opossum

ACNTATCVTHRLADFLSRSGGVGKNNFVPTNVGSKAF Chick

ACNTATCVTHRLADFLSRSGGMGNSNFVPTNVGAKAF Takifugu rubripes

GCNTSTCVTHRLADLLSRSGGLGYNNFVPTNVGAQAF Paralichthys olivaceus

ACNTATCVTHRLADFLNRSGGMGNSNFVPTNVGAKAF Oncorhynchus gorbuscha 1

ACNTATCVTHRLADFLSRSGGMGNSNFVPTNVGAKAF Oncorhynchus gorbuscha 2

ACNTATCVTHRLADFLSRSGGMGNSNFVPTNVGAKAF Tetraodon 1

ACNTATCVTHRLADFLSRSGGLGYSNFVPTNVGAQAF Tetraodon 2

ACNTATCVTHRLADFLSRSGGMGNSNFVPTNVGAKAF Takifugu 1

ACKTATCVTHRLADFLSRSGGLGYSNFVPTNVGAQAF Takifugu 2

ACNTATCVTHRLADFLSRSGGIGSSKFVPTNVGSQAF Danio

ACNTATCVTHRLADFLSRSGGIGSSKFVPTNVGSQAF Carassius auratus

ACNTATCVTHRLADFLSRSGGLGHSNFVPTNVGAQAF Oryzias latipes

Dark shading; identical amino acids. Species include representative mammals, marsupials (opposum), birds (chick) and bony fish (Takifugu, Paralichthys, Oncorhynchus, Danio, Carassius and Oryzias).

Supplementary Figure 2; N-terminal regions of CGRP/Calcitonin family members, aligned over CGRP_1-20_.

AC-DTATCVTHRLAGLLSRSG Human αCGRP

KC-NTATCATQRLANFLVHSS Human amylin

KC-NTATCATQRLANFLVRSS Rat amylin

KC-NTATCVTQRLADFLVRSS Chick Amylin

KC-NTATCVTQRLADFLVRSS Takifugu Amylin 1

KC-NTATCVTQRLADFLVRSS Tetroadon Amylin 1

SC-NTATCMTHRLAGLLSSAG Pig CRSP1

SC-NTASCLTHRLVGLLSRSG Horse CGRP1

SC-NTASCVTHKMTGWLSRSG Pig CRSP2

SC-KDGPCVTNRLEGWLARAE Dog CRSP2

SC-NTAICVTHKMAGWLSRSG Pig CRSP3

GC-RFGTCTVQKLAHQIYQFT Human AM

GC-RFGTCTMQKLAHQIYQFT Rat AM

GC-RFGTCTFQKLAHQIYQLT Mouse AM

GC-RFGTCTVQKLAHQIYQFT Dog AM

GC-RFGTCTVQKLAHQIYQFT Pig AM

GC-RFGTCTVQKLAHQLYQLT Chick AM

GC-SLGTCTVHDLAFRLHQL- Takifugu AM1

GC-SLGTCQVQNLTHRLFRLV Hagfish AMGC-VLGTCQVQNLSHRLWQLM Human AM2

GC-VLGTCQVQNLSHRLWQLV Rat AM2

GC-VLGTCQVQNLSHRLWQLV Mouse AM2

GC-VLGTCQVQNLSHRLWQLV Ferret AM2

GC-ALGTCQVQNLSHRLWQLV Cow AM2

GC-VLGTCQVQNLSHRLWQLV Pig AM2

GC-VLGTCQVQNLSHRLYQLI Oryzias latipes AM2

GC-VLGTCQVQNLSHRLYQLI Eel AM2

GC-VLGTCQVQNLSHRLYQLV Danio AM2

AC-VLGTCQVQNLSHRLYQLI Takifugu AM2

GC-FLGTCQVQNLSHRLYQLV Oryzias latipes AM3

GC-VLGTCQVQNLSHRLYQLI Eel AM3

GC-ALGTCQVQNLSHRLYQLI Takifugu AM3

GC-ALITCLYHDLVHLLHETN Oryzias latipes AM4

GC-NLATCSVHELAHLLNIMH Danio AM4

GC-ALFMCAYHDLLQRLNHIY Takifugu AM4

GC-FLFLCVHHNLLSRMEHFN Tetroadon AM4

LC-SLGTCQTHRLPEIIYWLR Pig AM5

GC-QLGTCQLHNLANTLYHIN Oryzias latipes AM5

GC-QLGTCQLHNLANTLYRIG Eel AM5

GC-QVGTCQVHNLANKLYQLG Takifugu AM5

GC-PLATCQTQNLANWLYLLA Squalus acanthias AM5-like

-CGNLSTCMLGTYTQDFNKFH Human Calcitonin

-CGNLSTCMLGTYTQDLNKFH Rat Calcitonin

-CSSLSTCVLGKLSQELHKLQ Danio Calcitonin

-CASLSTCVLGKLSQELHKLQ Chick Calcitonin

-CSNLSTCVLGKLSQELHKLQ Takifugu Calcitonin 1

-CAGLSTCVLGKLSQDIHKLQ Takifugu Calcitonin 2

-CSNLSTCVLGKLSQELHKLQ Tetroadon Calcitonin 1

-CAGLSTCVLGKLSQDIHKLQ Tetroadon Calcitonin 2

AM and AM2 both have significant N-terminal extensions, which are not shown; these do not appear to be important for biological activity. Dark shading; identical amino acids. Species include representative mammals, birds (chick), bony fish (eel, Takifugu, Tetroadon, Danio, and Oryzias), cartilaginous fish (Squalus) and jawless fish (hagfish).

Supplementary Table 1: Pharmacology of selected CLR/RAMP and CTR/RAMP complexes

| Receptor | CGRP | AM_1_ | AM_2_ | AMY_1(a)_ |
| --- | --- | --- | --- | --- |
| Composition | CLR/RAMP1 | CLR/RAMP2 | CLR/RAMP3 | CTR _(a)_/RAMP1 |
| Specificity | CGRP>AM≥AM2 | AM>AM2>CGRP | AM, AM2>CGRP | sCT ≥AMY,CGRP |

The CGRP, AM_1_ and AM_2­_ receptors have very low affinity for amylin (AMY); the AMY_1(a)_ receptor has very low affinity for AM and AM2. Further details may be found in the IUPHAR/BPS Guide to Pharmacology, http://www.guidetopharmacology.org/GRAC/FamilyDisplayForward?familyId=11
